# A Study of Sustainable Mortars Incorporating Copper Slag and Zeolites

**DOI:** 10.3390/ma19112302

**Published:** 2026-05-29

**Authors:** Santiago Rosado, Leticia Presa Madrigal, Luis Felipe Mazadiego, Juan Llamas, Lidia Gullón, Domingo Alfonso Martín, Jorge Luis Costafreda

**Affiliations:** 1 Universidad Polítecnica de Madrid, Calle Ríos Rosas, 21, 28003 Madrid, Spain; leticia.presa.madrigal@upm.es (L.P.M.); luisfelipe.mazadiego@upm.es (L.F.M.); juan.llamas@upm.es (J.L.); domingoalfonso.martin@upm.es (D.A.M.); jorgeluis.costafreda@upm.es (J.L.C.); 2 Fundación Gómez Pardo, Calle Alenza, 1, 28003 Madrid, Spain; direccion.tecnica@fgomezpardo.es; 3 Laboratorio Oficial para Ensayos de Materiales de Construcción (LOEMCO), Calle Eric Kandell, 1, 28906 Getafe, Spain

**Keywords:** copper slag, natural zeolite, mortar, cement, water demand

## Abstract

The generation of slag from copper smelting is between 40 and 45 Mt per year. However, the utilisation of this material in large-scale applications remains limited. This study evaluates the combined use of copper slag as a fine natural aggregate substitute (50, 75, and 100%) and natural mordenite-type zeolite as a partial cement replacement (25, 40, and 55%) in the development of sustainable mortars. The samples were characterised using XRF, particle size distribution, and density analysis, and sixteen mixtures were produced. The consistency and water demand in fresh state, the flexural and compressive strength at 7 and 28 days, and the porosity measurements of the hardened mortars were analysed. The results demonstrate that zeolite significantly increases water demand, leading to higher porosity (27–34%) and reduced mechanical strength (14–31 MPa). Conversely, copper slag decreases the water-to-cement ratio and produces denser matrices with the lowest porosity values (8–13%), achieving compressive strengths at 28 days that are higher (53–58 MPa) than the reference mortar (50 MPa). In hybrid mixtures, slag partially mitigates the porosity increase induced by zeolite, revealing a favourable interaction between both materials. Mixtures containing 75–100% copper slag and 25% zeolite (MZ-8 and MZ-9) exhibited balanced porosity (18–21%) and mechanical performance (38–39 MPa), confirming their suitability for mortar applications. The findings demonstrate that the joint incorporation of copper slag and natural zeolite is a viable strategy for producing eco-efficient mortars while reducing clinker and natural aggregate consumption.

## 1. Introduction

The production of different waste from the mining and metallurgical industry, which represents the 5–7% of the world’s GDP [1], is an important challenge today. For this reason, the technological innovation of the mining industry is focused on the recovery of these residues or, at least, on minimising waste. These actions are included in the concept of circular economy [2,3], which, in general terms, aims to optimise the production of primary and secondary raw materials by minimising energy consumption; reducing environmental impacts; recovering mining spaces; and reducing, treating, and reusing mining waste. Aware of these needs, the United Nations agreed to jointly address these efforts by setting the so-called Sustainable Development Goals (SDGs) [4], which aim to protect the environment while eradicating poverty and ensuring global prosperity. In addition, in the Climate Objectives Plan, the European Commission proposed to reduce greenhouse gas emissions by at least 55% below 1990 levels by 2030 and achieve climate neutrality by 2050 [5]. One of the strategies to mitigate carbon dioxide (CO_2_) emissions in cement plants is the use of Supplementary Cementitious Materials (SCMs) to reduce the amount of clinker present in the cement. According to data recorded by Getting the Numbers Right (GNR), the clinker factor was 83% in 1990, which was reduced to 76% in 2006, and it is expected to be reduced to 71% in 2050 [6]. This can save a large amount of fuel and reduce CO_2_ emissions, since clinker production consumes 3.70 MJ/tonne and emits 0.79 tonne CO_2_/tonne of clinker [7].

It is estimated that the current production of mining waste ranges between 5000 and 7000 Mt per year worldwide [8]. And the production of primary copper slags accounts for between 40 and 45 Mt per year [9,10]. For every tonne of pure copper produced, 2.2 tonnes of slag are generated as a by-product [11]. These slags are the result of the pyrometallurgical transformation of copper concentrates, while those from copper and scrap recycling are secondary slags [12,13]. From an economic perspective, the annual waste management costs associated with copper slag are estimated to be approximately 5000 M€ [14].

All this raises the need to provide new routes for the use of this by-product and avoid its deposit in landfills, which occurs in some parts of the world, where this waste does not have a massive industrial use. There are currently active reuse routes such as its use as an abrasive (if obtained as shot), its valorisation of different elements (such as Cu or Co) [15,16], an aggregate for construction materials (road pavements, fillings, bases and sub-bases, concretes and mortars), or a partial substitution in cements [17,18,19,20,21,22,23,24]. The use of these slags in cement-based construction materials can help mitigate the consumption of raw materials and CO_2_ emissions in this sector [11,25]. It is necessary to consider that the construction industry has an enormous consumption of raw materials. In Europe, approximately 25% of all raw materials are consumed by this industry [26], and this proportion is much higher in the case of fine aggregate in particular. The European construction sector consumes 85% of the sand produced [27]. On the other hand, one of the main challenges of the construction industry is the reduction in CO_2_ emissions. It is estimated that the cement industry produces approximately 5–8% of total CO_2_ emissions worldwide [28,29,30]. Between 50 and 60% of these emissions are due to the production of clinker [30], the main component of Portland cement, in which the decarbonation of calcium carbonate (CaCO_3_) into calcium oxide (CaO) and carbon dioxide (CO_2_) occurs.

Copper slags have already been used as aggregate in mortars and concretes with generally positive results [31] due to the strength of the slags themselves [32] and the angular shape of the particles, which allows for better adhesion between the paste and the aggregate [33]. In mortars, from low proportions to complete substitutions of fine aggregate with slags have been applied, obtaining compressive results at 28 days (21 MPa) similar to the reference value (18 MPa) [34], and even higher with 47.11 and 55.0 MPa for references of 32.7 and 22.5 MPa [34,35,36]. However, a more detailed study [35] with slag additions in different proportions showed that, although any proportion of slag addition results in an increase in the 28 day compressive reference value (24.6 MPa), the optimal point is reached for 50% with 42.7 MPa. In any case, substitutions of 40 and 60% are also interesting with 39.8 and 39.2 MPa.

The use of copper slag as fine aggregate in concrete has been extensively studied, although its effect on mechanical performance is not always consistent. Reported optimal replacement ratios vary depending on the type of concrete and its initial composition. For conventional concretes, the optimal substitution typically ranges between 20% and 50%, with compressive strengths comparable to or slightly higher than the reference mixtures [33,35]. However, in high-performance concretes, significantly lower replacement levels (around 10%) are often optimal [37], while higher proportions may lead to strength reductions [38]. These variations are mainly attributed to differences in w/c ratio and cement content, which strongly influence the effect of slag-induced free water and resulting porosity.

The different optimal substitution proportions in both mortars and concretes are explained by the low absorption of copper slags, which allows the presence of free water in the mixtures [37]. This free water generates porosity in the paste that surrounds the coarse aggregate particles [38] and the consequent decrease in strength. This is why at high proportions of slag, the final strength decreases despite being a stronger material [32].

As a partial substitute for cement, copper slags have also been used, although the results are much less promising due to two factors: first, the presence of heavy metals in the slags that interfere with the hydration reactions of the cement [19]; and second, the low proportion of calcium in the slags, which is the element that provides the cementing capacity [39]. In fact, the same study on three different slag samples [39] showed that the best behaviours were achieved for the highest proportions of CaO. In any case, it can be observed that the behaviour is acceptable up to 5% substitution, but above that, the values are very negative.

This negative behaviour makes the use of copper slags in cements less interesting than in aggregates, since the potential impact is much lower. In contrast, the combination of copper slags as aggregate and steel slags as an addition to cements has been studied with results above the reference value, even for a substitution of 40% of the aggregate and 20% of the cement [40]. Additionally, with 20% fly ash instead of cement and 40% copper slag per fine aggregate, similar values to the reference are reached [41]. And it has been shown that in concrete with silica fume as an addition to cement, copper slags can reach up to 40% proportion to obtain better results than with a natural aggregate [42].

The use of pozzolanic materials as SCMs can improve some properties of the original cement, such as impermeability, durability, and resistance to thermal cracking [43]. Among the pozzolanic materials used as SCMs are zeolites [44,45,46], hydrated aluminosilicates whose structure is formed by micropores, channels, and cavities. The main advantage of this material is its pozzolanic activity; that is, it reacts with Ca(OH)_2_ to form calcium silicates [47,48]. This alternative, using copper slags as a substitute for fine aggregate together with that of zeolites for cement in the manufacture of mortars, has not yet been tested. It has been shown that zeolites can be used in cements in a proportion of 25%, reaching compressive strength values of 39.6–41.5 MPa at 28 days (for a 42.5 MPa cement) [49], or even greater than 45 MPa at 28 days for a reference value of almost 60 MPa [50]. At these ages, the reference values of almost 50 MPa can be exceeded with calcination processes of the zeolites [51]. But even considering that the reference values are not reached, at later ages (180 days), mixtures with 10% zeolites that did not reach the reference value close to 50 MPa at 28 days exceed the reference reaching almost 60 MPa [52].

The present research aims to analyse the mechanical (nominal flexural and compressive strength) and physical (workability, water consumption, and density) parameters of mortars made with copper slags as a partial and total substitute for natural fine aggregate, as well as in addition with natural zeolites as a partial substitute for Portland Ordinary Cement. This combination of materials presents a novelty in the study of sustainable mortars that has not been studied and is of great interest, given that it aims to provide an alternative use for copper slags, reduce the consumption of raw materials in the construction sector and minimise emissions derived from cement production, thus achieving a sustainable mortar in line with the Sustainable Development Goals and the principles of the circular economy. However, the present study also has the objective of addressing a technical issue of some recrudescence in research, namely the increased porosity and reduced final strength that have been observed when high w/c ratios are employed. The present study examines how variations in w/c ratio associated with the use of slag and zeolite influence the fresh and hardened behaviours of mortars. While it is expected that zeolite increases porosity (high w/c ratio) and copper slag reduces it (low w/c ratio), the analysis of the combination of both is of interest. The data indicate that the excess water resulting from the low water absorption of the copper slag is absorbed by the zeolite, leading to an increase in porosity and a decrease in final strength. Notwithstanding the capacity of zeolite to adsorb water at its surface, the present research pertains to the concept of absorption, which is intrinsically associated with the manner in which water interacts with mortars. The research makes a direct contribution to understanding the relationship between w/c ratio, porosity, and final strength in materials that may be of great interest for the development of sustainable mortars.

## 2. Materials and Methods

In order to verify the possibility of the joint use of primary copper slags and zeolites, a number of representative samples were selected, and a testing plan was designed. This testing plan involved the manufacture of mortar specimens, the behaviour of which was then analysed in sustainable mortars. In total, 16 cement mortar mixtures were developed in which the cement was partially replaced by zeolites and the natural aggregate by copper slags. The cement used was CEM I (42.5 R) without any additions in order to evaluate the performance of the new materials. The natural aggregate was a CEN-normalised sand, as defined in UNE EN 196-1 [53]. It is a siliceous sand with a minimum silica content of 98%, and the particles are less than 2 mm in size. The water used was deionised [54].

The zeolites added to the cement have already been studied for similar purposes. These natural zeolites (ZEO) originate from the ‘San José-Los Escullos’ deposit in Almería, Spain. They primarily comprise natural mordenite (MOR), containing smectite and quartz impurities. The zeolites are rock fragments several centimetres in size that are light and white [50]. The pozzolanic activity of the sample has been previously documented [49]. This was determined through a comparison of the amount of calcium hydroxide present in a water solution (in contact with the hydrated cement) and the required amount of calcium hydroxide to maintain the alkalinity. The result was positive, as the amount of calcium hydroxide was found to be below the isothermal solubility at 40 °C.

Copper slag, produced by the Atlantic Copper Huelva Metallurgical Complex, primarily comprises iron silicate (Fe_2_SiO_4_) (>50%). Atlantic Copper’s iron silicate is a granular vitriol solid with a shiny black colour and is chemically stable. It presents two mineral phases: olivine with a concentration of 90% and an iron–silica oxide in the other 10% [55]. Despite the common nomenclature of copper slag, it should be noted that copper is a minor element.

Prior to testing, the zeolite required was prepared by crushing and milling with a ring mill (TS 1000 ring mill, Siebtechnick, Germany) to a size of less than 63 µm to ensure good reactivity with the cement. The slags did not require any preparation before being used in the cement-based mixtures.

**Figure 1:**
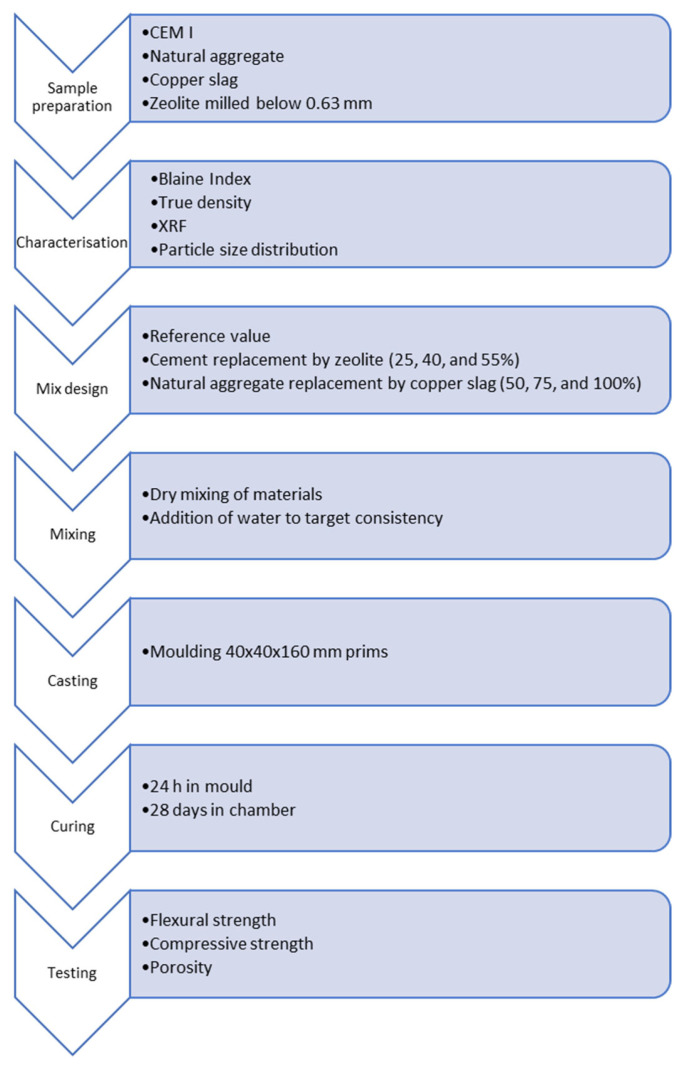
Summary of the methodology.

A schematic summary of the methodology is shown in Figure 1.

First, the Blaine specific surface is determined by UNE EN 196-6 [56] and the true density by an air pycnometer. The Blaine index is calculated by Equation (1):

(1) $$B.\;I.\;=\;\mathrm{Sref}\;\times \;\sqrt{\frac{t}{t_{ref}}}$$

where

*B. I.* is the Blaine index;

*Sref* is the specific surface of the reference material;

*t* is the time of testing;

*t_ref_* is the time of the reference material.

And the true density is calculated by Equation (2):

(2) $$d=\frac{m_{s}}{m_{s}+m_{p}-m_{sp}}$$

where

*d* is the true density;

*m_s_* is the mass of the sample;

*m_p_* is the mass of the pycnometer;

*m_sp_* is the mass of the pycnometer containing water.

The composition of each of the materials used was analysed to study the potential reactions that may occur with the cement and aggregate. The samples were melted into a pearl for characterisation using dispersive wavelength X-ray fluorescence (XRF) on a BRUKER S4 PIONEER instrument (Bruker, Middlesex, NJ, USA) with a detection limit of 0.01% for Al, Ba, Ca, Cr, Fe, K, Mg, Mn, Na, P, S, Si, Sr, and Ti oxides. Calcination losses were performed at a temperature of 1000 °C.

The analysis of the particle size distribution (PSD) of each waste was conducted in accordance with the UNE EN 933-1 standard [57]. The materials were subjected to a manual method involving horizontal and vertical circular motion for a duration of at least five minutes per sieve, or until the mass change in the material retained was less than 1% per minute. The materials were tested in a dry state and without prior washing. In accordance with the specified methodology, sieves with openings ranging from 2 mm to 63 µm were employed in the following sizes: 2 mm; 1 mm; 0.50 mm; 250 µm; 125 µm; and 63 µm. The mass of the material retained in each sieve was determined using a balance (Melter Toledo) with an accuracy of 0.01 g.

The dosage and manufacture of the mortars followed the standard UNE EN 196-1 [53], with a cement-to-fine-aggregate ratio of 1:3 (25% of cement and 75% of fine aggregate). The cement was partially replaced by zeolite in three different proportions: 25%, 40%, and 55%. With the slags, the natural aggregate was replaced in three other proportions: 50%, 75%, and 100%. Additionally, combinations of the two substitutions were created, resulting in 15 total mixtures and an additional reference mixture with no additions. Table 1 shows the proportions of the mixtures.

Since the water absorption of the developed mixtures was unknown, the amount of water needed for each mixture was determined experimentally. For this purpose, water was added until a consistency value of 185 ± 5 mm was achieved. This test was conducted in accordance with the UNE EN 1015-3 standard [58], which delineates the flow table test. A truncated conical mould with a base diameter of 100 mm was utilised, and it was filled with fresh mortar in two layers. The mould was then positioned at the centre of the flow table. Subsequent to the removal of the mould, the table was subjected to 15 vertical impacts (jolts) at a rate of one per second. The resulting spread of the mortar was measured along two perpendicular diameters using a calliper, and the average value was recorded as the flow value (mm). It should be noted that water demand in this study was determined indirectly through the consistency test, rather than by direct absorption measurements. This approach was intentionally adopted, as it reflects the effective water required to achieve comparable workability under standard conditions. Therefore, the obtained values represent the practical behaviour of the mixtures, integrating both absorption and surface-related interactions, rather than an isolated material property.

Flexural and compressive strength were analysed at 7 and 28 days according to the UNE EN 196-1 [53]. Three prismatic specimens measuring 40 × 40 × 160 mm were made for each age (7 and 28 days). Three were tested for flexural strength, while compression testing was performed on the six pieces resulting from the flexural test. Only the final average values for each type of measurement are shown. The formula used to determine the flexural strength is:

(3) $$S_{f}=\frac{3\times F\times L}{2\times b\times h^{2}}$$

where

S_f_ is the flexural strength;

*F* is the fracture load;

*L* is the span between supports;

*b* and *h* are the width and height of the prism (mm), respectively.

Equation (4) is used to determine the flexural strength:

(4) $$Sc=\frac{F}{A}$$

where

*Sc* is the compressive strength;

*F* is the fracture load;

*A* is the loaded area.

Porosity has been calculated based on the results obtained from the water absorption test, which was carried out in accordance with the ASTM C642-21 standard [59]. To this end, a number of samples were extracted from each of the mortars examined at 28 days, and were then placed into individual containers for an initial period of 48 h until it was ascertained that saturation had been achieved. The drying process was conducted at a temperature of 100 °C for a duration of 24 h until a constant mass was achieved. The results are expressed individually and in relation to the compressive strength at 28 days. The formula used is given as Equation (5):

(5) $$P=\frac{m_{s}-m_{d}}{m_{s}-m_{i}}\times 100$$

where

P is the porosity;

*m_s_* is the saturated mass;

*m_d_* is the dry mass;

*m_i_* is the immersed mass.

As the behaviour of the materials was previously unknown, it was necessary to ensure that the specimens had been manufactured correctly. To this end, the density of the hardened mortar mixtures was evaluated using a variation of the UNE EN 1015-10 standard [60]. The mass of the hardened but not dried mixtures was determined 72 h after mixing. The volume was estimated based on the dimensions of the mould and specimen. The aim was not to obtain an exact density value but rather an additional value that enables the manufacture of the specimens to be monitored. The density formula is given as Equation (6):

(6) $$D=\frac{m}{V}$$

where

D is the density;

*m* is the specimen mass;

*V* is the volume of the mould.

## 3. Results

### 3.1. Characterisation

The physical properties of the materials were also determined. The cement presented a Blaine specific surface area of 4000 cm^2^/g and a true density of 3.08 g/cm^3^. The natural aggregate showed a true density of 2.59 g/cm^3^. The zeolite exhibited a lower density (2.34 g/cm^3^) and was prepared with a higher fineness than the cement, with a Blaine specific surface area of 4500 ± 200 cm^2^/g. In contrast, copper slag presented the highest density among all materials (3.91 g/cm^3^), suggesting a potential influence on the compactness of the resulting mixtures.

The composition of the materials analysed by X-ray fluorescence is shown in Table 2. The presence of alumina (Al_2_O_3_), calcium oxide (CaO), iron oxide (Fe_2_O_3_), and silica (SiO_2_) is notable, as these are the compounds with the highest proportions. In the case of zeolite, these compounds could provide information about the mechanical behaviour of the final mixture. Additionally, the significant calcination losses indicate the presence of organic matter or volatile compounds.

It is possible to highlight the negative value of calcination loss. This was very likely due to the oxidation of some metals causing a weight gain, which occasionally occurs with some slags [61].

**Figure 2:**
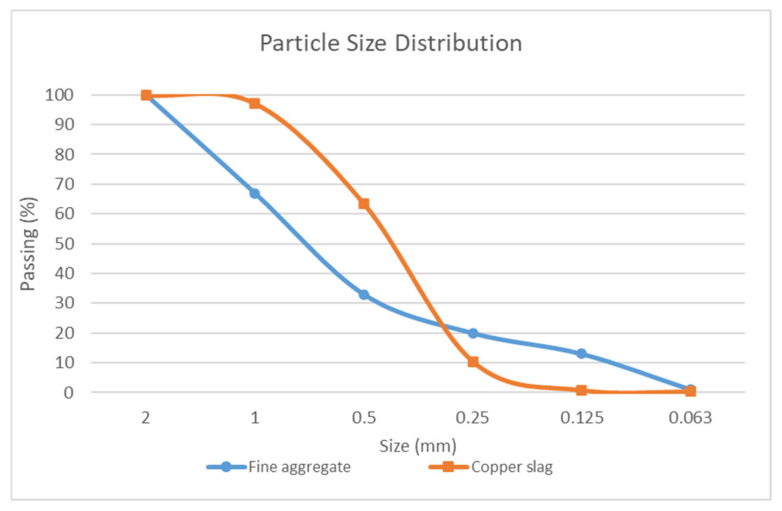
Particle size distribution of aggregates.

As illustrated in Figure 2, the objective of the PSD is twofold. Firstly, it seeks to ascertain the fundamental characteristics of the aggregates utilised. Secondly, it aims to evaluate the potential for compaction of the aggregates.

It has been observed that fine natural aggregate exhibits a homogeneous distribution, attributable to its standardised composition. Conversely, copper slag is characterised by a low proportion of particles measuring less than 0.25 mm, a consequence of its industrial origin. The fineness modulus (FM) was calculated as 2.67 for the natural aggregate and 2.28 for the copper slag, indicating a finer overall grading of the slag. This result complements the PSD analysis, confirming differences in particle distribution and supporting the observed compaction behaviour.

### 3.2. Water Consumption

**Figure 3:**
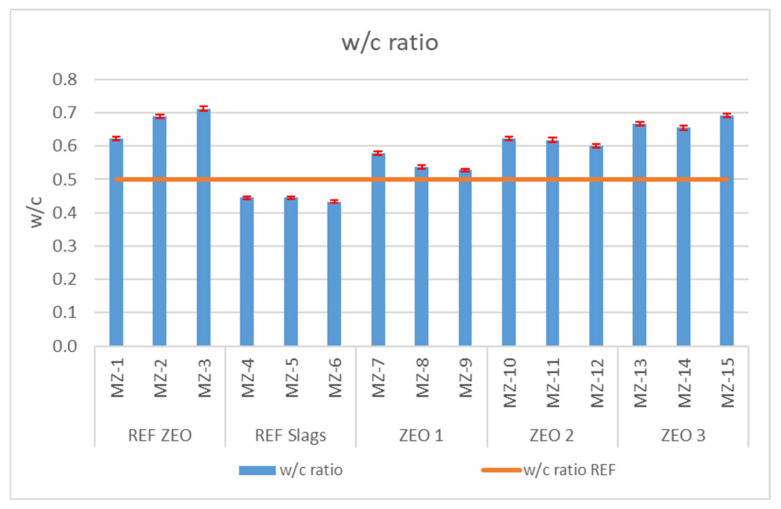
Water consumption as w/c ratios.

**Figure 4:**
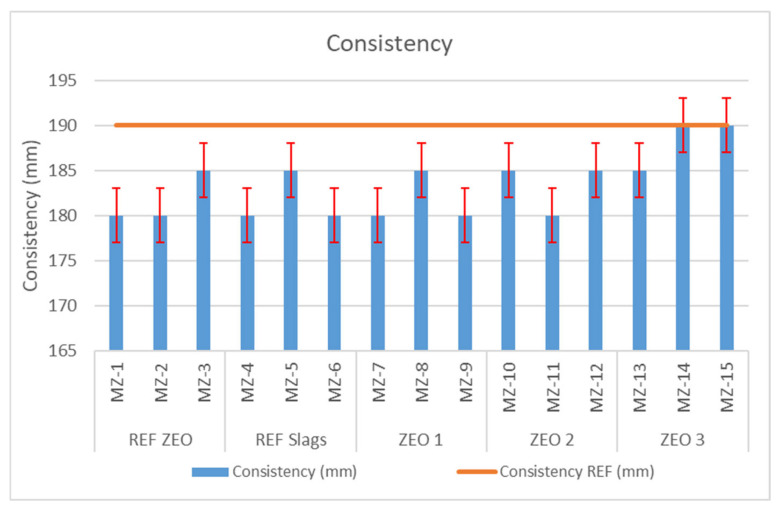
Water consumption as consistencies.

The water-to-cement ratio (w/c) provides a direct indication of the potential mechanical behaviour of different mixtures [62]. Although the most positive values in terms of strength are achieved at low w/c ratios, a compromise is necessary in terms of fluidity or workability. For this reason, the consistency value of all the mixtures is located between 180 and 190 mm, with the minimum amount of water required to achieve this value. These results are shown in Figure 3 and Figure 4.

Interestingly, it is practically impossible to maintain a consistent value of consistency between 180 and 190 mm. However, a value always below 190 mm was maintained to ensure that excess water does not reduce the final strength. Conversely, there was a significant increase in water demand, exceeding the reference value of 0.5, except in the case of slags. These two aspects suggest that new materials affect the water consumption of mixtures differently: slags reduce water consumption, while zeolite increases it. Therefore, considering only the effect of water, mixtures containing slags should be stronger, whereas those containing zeolite will have a lower mechanical strength.

### 3.3. Mechanical Strength

The following figures illustrate the flexural and compressive behaviour of the mixtures. The good performance of the slags was evident (REF slags), as they matched or even exceeded the reference value (REF). Conversely, mixtures containing zeolite (REF ZEO) as a partial cement substitute exhibited significantly lower strength values than the reference (REF), especially for high substitution ratios (MZ-3). This reduction in mechanical strength can be further interpreted by considering both the microscale and nanoscale. The overall mechanical performance was strongly influenced by the microstructural architecture of the mortar, including porosity [63]. At the nanoscale, modifications in the binding phase and pore structure induced by zeolite incorporation may weaken the stiffness and cohesion of the matrix [64].

**Figure 5:**
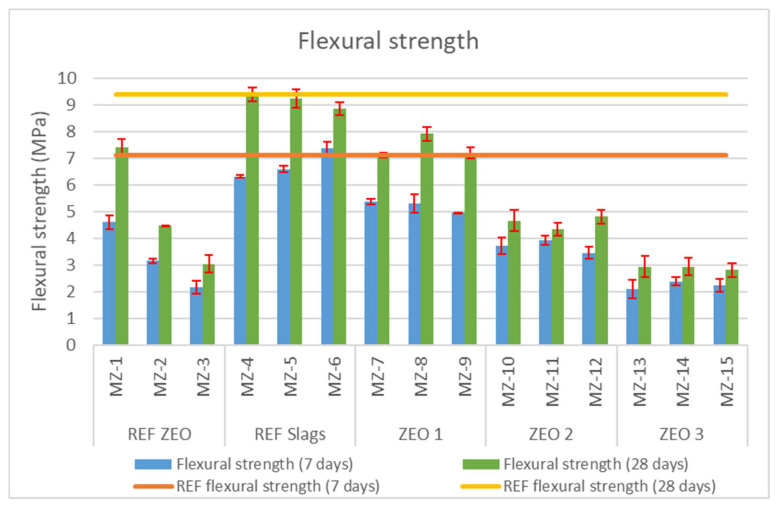
Flexural strength at 7 and 28 days.

The flexural results (Figure 5) showed that only the REF slag samples reached the reference values at 7 and 28 days (7.1 and 9.4 MPa, respectively), which are indicated by two horizontal lines.

The flexural strength at 7 and 28 days can be seen for each of the mixtures. It is noteworthy that behaviour was not always symmetrical (a low value at 7 days does not imply a low value at 28 days). This can be seen in the REF slags group, where the strength increased at 7 days but decreased at 28 days. No clear correlation was observed in the other groups. However, the strength decreased with increasing zeolite content (REF ZEO). Generally, the results were below the reference values, although the REF slags group was comparable.

**Figure 6:**
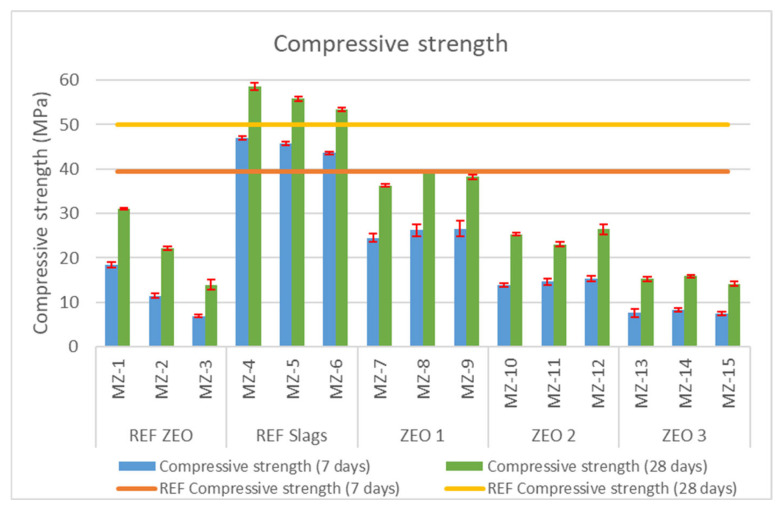
Compressive strength at 7 and 28 days.

A similar pattern emerged for compressive strength (Figure 6), where only the REF slags samples reached (and exceeded) the reference values of 39.4 and 50.0 MPa at 7 and 28 days, respectively, as indicated by the horizontal lines. This clearly indicates that using slags improves compressive strength, while using zeolite worsens it.

However, in this case, the symmetry between the 7 and 28 day results have shown a clearer trend. In general, the behaviour at 7 days allows the 28 day strength results to be predicted. There are, however, specific cases where this is not the case, such as in the comparison of MZ-10 and MZ-11. The latter exhibits better performance at 7 days but worse performance at 28 days than MZ-10.

### 3.4. Porosity

**Figure 7:**
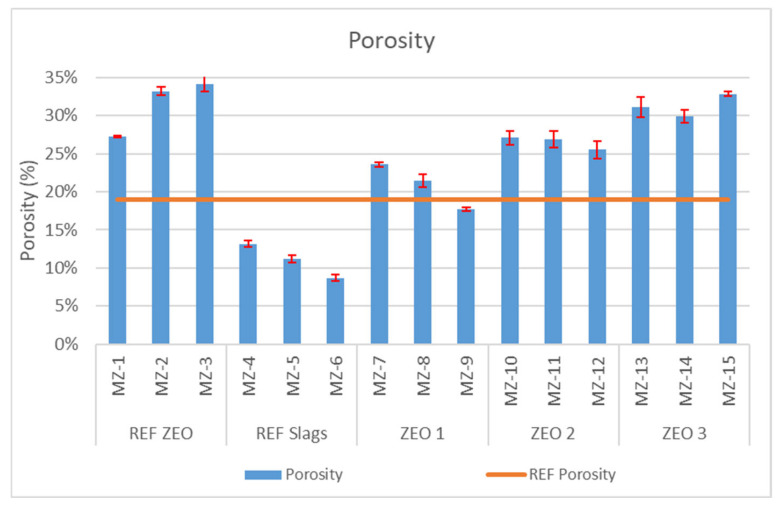
Porosity of hardened mortars.

As illustrated in Figure 7, the porosity outcomes of the hardened mortars are presented. It is evident that zeolites generally promote elevated porosity levels, while copper slags contribute to reduced porosity compared to the reference mixture. This directly governs the mechanical strength.

### 3.5. Density

**Figure 8:**
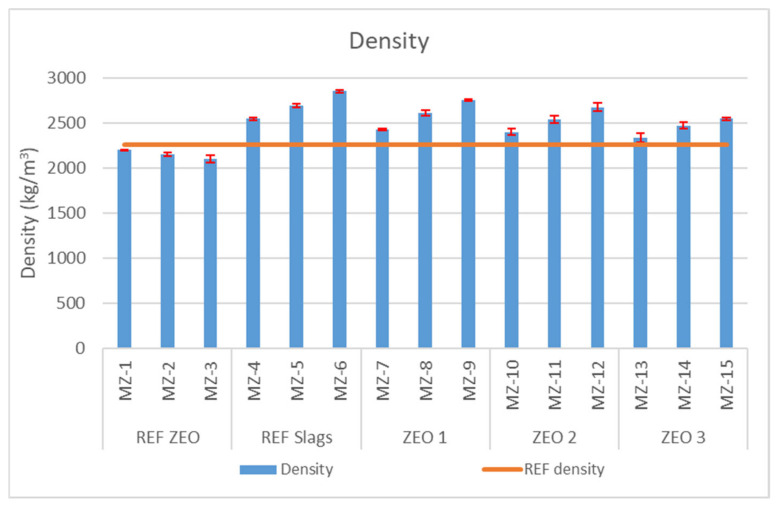
Density of hardened mortar.

Figure 8 shows the density values of hardened mortar in relation to the reference value (the horizontal line).

It was observed that copper slag, which has a higher density than the normalised aggregate, increased the density of the mixtures. In contrast, zeolite, which is lighter than cement, decreased the density of the final mixtures. In any case, these results demonstrate homogeneous behaviour in all groups and thus verify that the specimens were manufactured correctly, which was the objective of this test. A significant increase in density, as produced by slags, resulted in an increase in mechanical strength [65]. In fact, the denser the mixture, the greater its strength.

## 4. Discussion

### 4.1. Characterisation

The cement has a typical composition for this type (CEM I) [66,67], with a high calcium content (>60%) and silica content (19.86%), as well as notable concentrations of aluminium, iron, potassium, and magnesium. The sulphur content likely originates from the gypsum used alongside the clinker in cement production and acts as a setting regulator [68].

In the zeolite, silica (SiO_2_) predominates with high values, while alumina (Al_2_O_3_) is reduced compared to other natural zeolites [69,70,71]. Calcination losses are somewhat higher than in other materials, indicating the presence of water molecules. Although the silica and alumina content directly affect pozzolanic reactions, small amounts of materials that are not zeolites normally coexist in natural zeolites and may or may not have pozzolanic properties. Therefore, predicting the pozzolanic behaviour of zeolites based solely on their silicon and aluminium content is complex [72]. In any case, the use of this zeolite at a proportion of 25% as an additive to Portland cement has previously been studied, demonstrating its pozzolanic activity [49].

The normalised sand contains a high proportion of silica (SiO_2_), although this is lower than the standard requirement of 98% for the manufacture of normalised mortars [53]. This small difference may be due to various factors, such as the analytical method used or contamination of the sample. In any case, this does not seem significant for the purpose of this study.

The slags show a clear predominance of iron (Fe_2_O_3_) and silica (SiO_2_), due to the high presence of iron silicate (>50%) and low proportions of alumina (Al_2_O_3_), calcium oxide (CaO), and sulphates (SO_3_). The iron (64%) content in particular, as well as the silica (30%) content, is high compared to the content of slags from other works (12–64% and 10–59%) [12,25,34,35,36,38,73,74]. The high strength of iron silicate makes it predictable that the slags will have a higher performance than natural aggregates in terms of mechanical strength of the hardened mixtures. The relatively high presence of sulphates (1.44%) is also noteworthy given the values reported in the literature (0.15–1.89%) [12,25,34,35,36,38,73,74]. Although this could lead to durability issues, the low proportion does not prevent its use. In fact, it has been found that using copper slag improves durability in terms of resistance to sulphates [75]. Finally, the low amount of calcium (1.32%) compared to values reported in other studies (0.05–8.84%) suggests that the slags under study will not develop cementing activity [39].

Fine natural aggregate and copper slag have been found to demonstrate similar particle size distribution (PSD), with particles measuring below 2 mm with an absence of fineness. This is considered a positive outcome, given that fines typically exhibit a higher water consumption in comparison to larger particles [76].

Nevertheless, the most significant characteristic that must be analysed is that of the compacting capacity. It is evident that the presence of smaller particles in copper slag, relative to those found in the natural aggregate, results in an increase in compactness. This, in turn, leads to the development of a superior mineral skeleton in comparison to that of the natural aggregate [34]. It is imperative to emphasise the pivotal role of this process in final mechanical strength development. The optimisation of this process will be achieved through the integration of both materials as opposed to the complete replacement of natural aggregate.

### 4.2. Water Consumption

Notably, that the w/c ratio increases with the amount of zeolite added. This is due to the nature of zeolite, specifically mordenite. The greater the amount of zeolite, the greater the water demand required to achieve a similar consistency, which will result in a decrease in final strength driven by an increase in the porosity.

The increased water demand in concrete and mortar mixtures containing zeolites is well-known due to the high absorption of natural zeolites [77], which is due to their microporous structure and high surface area [78]. This effect is further enhanced by the lower density of zeolites relative to cement, resulting in an increased volume of the final cementitious material. Ideally, the w/c ratio would be slightly increased to ensure a suitable mixture is achieved. It is evident that elevated water consumption has a deleterious effect on the final products, given that it results in an increase in the final porosity. This has a significant impact on the development of the mechanical strength of the final products. This disadvantage can be mitigated by using superplasticisers, although their high consumption can make the use of zeolites more expensive than using Portland Ordinary Cement [78]. Nevertheless, this option could be interesting, as it reduces CO_2_ emissions from cement production primarily through the significant increase in final strength.

In the case of slags, water consumption remains constant and below the reference value regardless of the proportion used. In this instance, it is imperative to consider the significantly higher density of copper slag in comparison to that of natural aggregate. Consequently, the volume of aggregate in MZ-6 is reduced relative to that in REF, thereby enabling the utilisation of lower w/c ratios. The lower water consumption in mixtures with slags is also due to this material’s low water absorption, which is due to its vitreous surface [37]. These offer a significant advantage, as it is possible to work with smaller amounts of water (thereby saving water, decreasing the porosity, and increasing strength) than with natural aggregates. Additionally, the presence of free water, which improves workability, could reduce the need for superplasticisers in concrete [79]. This is particularly important given that recycled aggregates currently in use generally have a higher water consumption than natural aggregates [76,80].

Slags reduce the enormous water consumption of zeolite-containing mixtures. This can be observed by comparing the water consumption of the MZ-1 and MZ-3 samples (with different zeolite proportions and no slags) with the following groups (incorporating both zeolite and slags): MZ-1 vs. ZEO 1; MZ-2 vs. ZEO 2; and MZ-3 vs. ZEO 3.

In all three cases, the groups incorporating slags have a lower water consumption than the groups that do not. One might think that this effect is maximised for the largest amounts of slag (MZ-9, MZ-12, and MZ-15), but no direct relationship is observed. As will be demonstrated subsequently, this phenomenon can be attributed to the absorption of excess water by the zeolite. Indeed, the most erratic behaviour is observed in ZEO-3, which contains the highest amount of zeolite. This is directly related to porosity and explains why ZEO-3 does not exhibit high mechanical strength. In any case, it can be concluded that the presence of zeolite is decisive in terms of water demand, despite the associated decrease in water consumption when slags are used. The outcome of these processes is an increase in porosity, which consequently reduces the final strength of the products.

### 4.3. Mechanical Strength

In general, the flexural strength of the mixtures behaves very similarly to the compressive strength. However, at 7 days, the REF slag exhibits inverse behaviour in terms of flexural and compressive strength. While flexural strength increases with the proportion of added slag, compressive strength decreases. The same downward trend occurs at 28 days with an increase in the proportion of slags, in both compression and flexural strength. There does not appear to be an obvious reason for this behaviour, although it should be noted that the differences in flexural strength are not very high (at most 1 MPa). Furthermore, in the other groups, the proportion of slag used is not the most decisive factor in the achieved strength.

Even for the smallest addition of zeolite (25%) in the REF ZEO (MZ-1), the strength decreases notably compared to the reference. However, for the highest additions of zeolite, the loss of strength is much more significant. However, it should be noted that the strength development in zeolites is slower, so it would be advisable to evaluate these mixtures in the long term. In any case, greater strength could be expected at 28 days for the 25% mixtures based on the literature [49,81]. In fact, under similar conditions and using the same zeolite sample, strengths higher than 40 MPa were achieved in previous studies [49]. The difference in strength between this study and previous ones lies in the w/c ratio (0.62 in this study versus 0.50 in [49]), which is due to high water consumption of zeolite-containing samples [78]. While a fixed w/c value was used in the previous work, this study has set a minimum workability. Therefore, the water demand is a critical parameter whose variation directly affects the porosity and the mechanical strength. However, it is important to highlight why the w/c ratio based on consistency is considered beneficial in this research. On the one hand, materials with very different densities and highly ambitious replacement ratios are being used. It is evident that reducing the w/c ratio to fixed values would result in alterations to the conditions of the mixtures in their fresh state. Consequently, consistency has been selected as the fixed parameter. Conversely, it is imperative to establish a discussion on the absorption of the excess water from the copper slag by the zeolite. This aspect is considered to be of pivotal significance to this research; it could not be evaluated in any other way. This hypothesis will be demonstrated in the next sections; however, the requirement for future research is to implement strategic water management in order to optimise the w/c ratio with the requirements of all materials and with the porosity. This will ensure the optimisation of final mechanical performance. Conversely, it is evident that the fineness of the zeolite influences its reactivity. Although higher degrees of milling will favour reactivity and will improve final mechanical strengths, for the same sample and the same Blaine index, both reactivity and pozzolanic activity have been demonstrated [49]. In a similar manner, the gradual development of pozzolanic materials enables the material to develop additional strength beyond 28 days. The discrepancy between the mechanical results obtained and those reported in the literature confirms that pozzolanic reactivity alone is not the governing factor. Instead, the adopted mixture design strategy is found to be a primary factor, with the water demand control approach proving particularly significant.

In the case of slag, relatively consistent behaviour is obtained regardless of the proportion used. However, in the REF slag group, the optimal mixture incorporates 50% slag. This is due to two factors: First, the slag allows for better compaction of the aggregate. The characterisation Section 4.1 provides a comprehensive demonstration of the manner in which slag complements natural aggregate in terms of PSD. The observation that the former possesses a smaller particle size than the latter enables its incorporation between the natural aggregate particles, thereby reinforcing the mineral matrix of the hardened mortars. Second, the slag leaves free water [79] that reacts with the cement-zeolite mixture. It is therefore reasonable to assume that mixtures with a higher proportion of slag will have slightly lower strength. For this group, greater strength is achieved than those with natural aggregate, thanks to the high strength of slag [82] and the fact that it absorbs less water than natural sand [37,74]. Although an increase in mechanical properties was expected, when 100% slag (MZ-6) is used, the increase in compressive strength after 28 days is lower than in other studies. This may be due to the use of lower strength cement, which allows the role of the slag to be more decisive. However, Al-Jabri et al. [74] obtained a similar increase for the optimum mixture of 50%, though the difference in strength was notable. However, a lower strength cement was also used.

No clear trend is observed for samples incorporating zeolite as a function of the proportion of slag added, since the free water produced by the slag is consumed by the zeolite (increasing the porosity). For ZEO 1 mixtures, MZ-7 has the lowest strength and the highest water consumption. MZ-8 and MZ-9 mixtures have very similar water consumption, but MZ-9 is slightly weaker. Similarly, in ZEO 2 mixtures, MZ-10 has a higher strength than the MZ-11 mixture for the same water consumption. MZ-12 has slightly lower consumption and slightly higher strength. This effect is not noticeable in the ZEO 3 mixtures, where strength is lower for higher water consumption (MZ-15). This is the first indicator that free water is absorbed by the zeolites, which would otherwise result in a consistent downward trend in the ultimate strength of the samples containing both materials. However, the behaviour is irregular, proving that there is a variation in porosity (which will be assessed in the next section) due to this increase in free water due to the presence of the slag. This phenomenon results in minimal differences in strength for the same proportions of zeolite and different proportions of slag (less than 15%). It is consistent with the observed macroscopic trends in water demand, porosity, and mechanical performance, as well as with mechanisms reported in the literature for porous supplementary cementitious materials.

In general, the trend for zeolite mixtures at 7 days is the same than at 28 days depends on the substitution ratio. That is, the greater the amount of zeolite, the lower the strength. However, there are slight differences in slag between the two ages. Beyond this change in behaviour, the values are considered to be very similar.

While zeolite decreases the strength of mortar mixtures, it is necessary to determine the Resistance Activity Index (RAI) using a method similar to that employed in UNE EN 196-1 [53] in order to understand if zeolite addition is active or not. This index stipulates that, for cement replacements of 25%, the compressive strength after 28 days must be at least 75% of the reference value. Likewise, 40% and 55% replacements can be analysed in the same way, setting the strength value at 60% and 45%, respectively.

**Figure 9:**
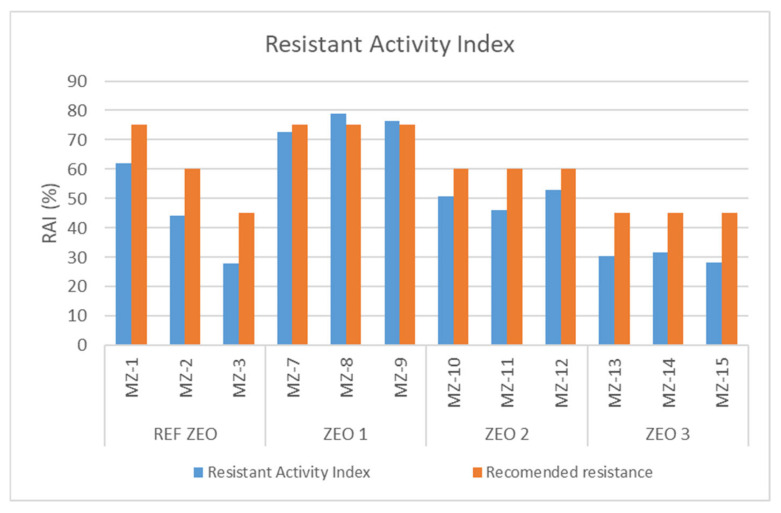
RAI of zeolite-containing mixtures.

Figure 9 shows the compressive strength index for each mixture (excluding MZ-4 to MZ-6, which do not contain zeolite). Only mixtures MZ-8 and MZ-9 have a compressive strength higher than the required value. Higher zeolite additions result in lower RAI values, as demonstrated by the comparison between MZ-2 and MZ-3. However, slag compensates for this decrease, which is why the MZ-8 and MZ-9 mixtures, which contain lower amounts of zeolite and higher amounts of slag, meet the recommended values. This effect serves to reinforce the prevailing theory of the free water sequestration by the zeolite. Despite the absence of indications of pozzolanic activity in the present results, this activity has been previously demonstrated [49]. The decrease in RAI with an increase in zeolite proportion is explained by the w/c ratios used, which are well above 0.5. This excess water increases the porosity, which decreases the strength of the test specimens, regardless of the pozzolanic activity. For this same reason, mixtures with slag have values closer to the recommended ones (due to lower porosity rather than the resistant properties of slag).

### 4.4. Porosity

**Figure 10:**
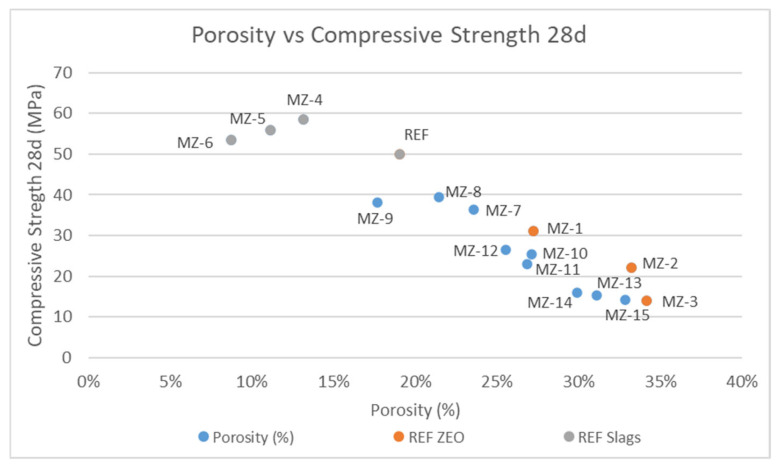
Relationship between porosity and mechanical performance.

As demonstrated in Figure 10, the porosity of the mortar, in conjunction with the properties of the materials utilised, exerts a significant influence on its mechanical performance. This relationship is evident in the correlation between porosity and the mechanical performance of the mortar. The direct relationship between porosity, the capillary connectivity, and the compaction grade of the matrix facilitates the interpretation of the differences between the mixtures.

A systematic analysis of the results reveals a clear inverse correlation between effective porosity and mechanical performance. The increase in total porosity, driven by the higher water demand of certain admixtures, acts as the primary determinant for the reduction in compressive strength. This behaviour is governed by water distribution within the system, which ultimately determines the capillary network and the effective porosity of the hardened matrix.

The REF ZEO group exhibited the highest porosity values, ranging between 27% and 34%, which directly accounts for its lower mechanical strength. This phenomenon is attributed not to a clinker dilution but instead to a physical mechanism. In order to maintain the target consistency in the samples with zeolites, it is necessary to employ a high w/c ratio. The intrinsic porous structure of the zeolite particles promotes high water absorption [83]; once this water evaporates during the hardening process, it leaves behind an interconnected capillary network that increases total porosity and reduces the effective load-bearing cross-section [84]. These findings align with the established theory that while zeolites possess pozzolanic reactivity, the physical effect of the increased w/c ratio can override the chemical benefits if not strictly controlled.

Conversely, the REF slags group demonstrated the highest mechanical strength, corresponding to the lowest porosity levels recorded (8–13%). This behaviour is justified by the low water absorption and vitreous surface texture of the copper slag particles, which allow for a significant reduction in the w/c ratio compared to both the reference and zeolite-bearing mortars. This promotes a more compact microstructure and a denser Interfacial Transition Zone (ITZ) [85,86].

In hybrid mixtures (ZEO-1, ZEO-2, and ZEO-3), the zeolite remains the dominant factor influencing the overall porous structure. However, a synergistic effect is observed: the presence of slag partially mitigates the negative impact of the zeolite. Notably, samples with the highest porosity levels in the study were those lacking slag. The decrease in porosity with copper slag is further enhanced by the particle size of the slag, which allows for a more compact mineral matrix. However, this is not the sole determining factor for the complete replacement of natural aggregate. As previously mentioned, as well as observed in the PSD, a combination of both materials is necessary to optimise the results in terms of density, porosity, and final mechanical strength.

Specific anomalies in the trend warrant further discussion. For instance, MZ-9 exhibited a strength similar to MZ-7 and MZ-8 (36–39 MPa), despite showing lower porosity (17% vs. 21–23%). This suggests that at this specific substitution level, the water distribution within the matrix is governed by the zeolite’s internal absorption, limiting the development of additional hydration products that would otherwise enhance strength. Furthermore, mixtures MZ-1 and MZ-2 deviated from the general trend by maintaining relatively high strength despite elevated porosity. This supports the hypothesis that the excess water released by the slag is effectively sequestered by the zeolite, an aspect that is supported by other works [84,87,88]. This phenomenon indicates that strategic water management and the optimisation of zeolite–slag ratios could lead to superior mechanical performance in future sustainable binders [89,90].

The substantial rise in water demand that accompanies zeolite incorporation represents a key challenge for its practical implementation. Merely reducing the w/c ratio is not the optimal solution, as it results in mixtures that are excessively dry, with suboptimal workability, incomplete compaction, and limitations in mechanical performance. The incorporation of superplasticisers can enhance fresh state properties to a certain extent; however, it can also lead to highly cohesive and adhesive mixtures, which may pose challenges in terms of handling and processing at an industrial scale, without achieving the optimal strength values. From an engineering perspective, these limitations are not merely theoretical but represent practical constraints that hinder large-scale applicability, especially due to difficulties in mixing, casting, and compaction. Alternative strategies, such as pre-saturating zeolite particles, could assist in mitigating water absorption during mixing; nevertheless, this approach necessitates further experimental validation. These findings underscore the importance of regulating the zeolite replacement level to achieve a balance between workability, porosity, and mechanical performance. Based on the current outcomes, replacement ratios should be confined to 25% or lower, in conjunction with the application of superplasticisers and the pre-saturation of the zeolite, to ensure acceptable engineering properties.

### 4.5. Density

The density of all the mixtures shows the same trend. Slag increases density, while zeolite decreases it. There are no values that deviate from this trend, demonstrating that there were no manufacturing errors.

Density can be used as a final point of discussion to verify the main hypotheses previously put forward regarding the determining factors of water consumption by fresh mixtures, the porosity of the final products, and the mechanical strength obtained. A triad of specific aspects have been identified concerning zeolite, copper slag, and the relationship between the two, as well as the final water not absorbed by the copper slag.

As the density of zeolites is lower than that of cement [91,92,93], the density of the mixtures decreases in the presence of zeolite. This implies that the proportion of zeolite by volume is higher than by weight. However, density does not appear to be a determining factor in terms of strength; fineness and water content are more critical aspects. The finding that REF ZEO exhibits the lowest density, especially when contrasted with the lower density of the ZEO-3 group relative to the equivalent mixtures in the ZEO-1 and ZEO-2 groups, unmistakably signifying a rise in porosity levels (as has previously been documented). It is therefore concluded that the final strength is not a consequence of the quality of the zeolites; instead, it is due to the increase in the w/c ratio.

Conversely, slag has a significantly higher density than natural aggregate [94,95], so it was to be expected that mixtures containing more slag would be denser. This has a positive effect on strength development, and the use of high-density aggregates is indeed common practice in the production of high-performance concretes [65]. The REF slags group exhibits the highest density, and that the samples with the highest slag content are also those with the highest density in each sample group. However, the fact that porosity and final strengths do not show such consistent behaviour indicates that material composition is not the only fundamental factor. On the one hand, there is the water content; however, given that REF slags have a similar w/c ratio but different porosity and final strength, it can be concluded that the particle size distribution of the slag complements the natural aggregate up to an optimised substitution ratio of 50%.

Finally, it has been theorised that zeolites sequester the water that the slags do not absorb. ZEO-1 not only exhibits a higher density than ZEO-2 and ZEO-3 but also a considerably lower porosity, higher strength, and a higher resistant activity index. This finding indicates that the excess water from the slag is absorbed by the zeolites, resulting in the formation of a larger capillary network as their presence increases. This process leads to enhanced porosity and a reduction in final mechanical strengths.

## 5. Conclusions

Based on the tests and analyses carried out on the materials under study—zeolite (mordenite) and copper smelting slag—and the detailed discussion of these, several interesting conclusions can be drawn.

The results demonstrate that the governing mechanism was not the intrinsic reactivity of zeolite or the mechanical quality of slag but the redistribution of mixing water between both materials, which controlled the effective porosity of the hardened matrix. This is the principal contribution of this work: the identification of water management as a primary concern rather than material substitution alone.

The combination of copper slag as an aggregate substitute and natural zeolite as a cementitious additive represents a technically viable approach to the development of sustainable mortars. The MZ-8 and MZ-9 mixtures, which contain 25% zeolite and either 75% or 100% copper slag, are suitable for the purposes of this study. These mixtures were achieved with minimal strength loss, high reactivity (IRA), and very high proportions of recycled material with high availability.

The mechanical and physical behaviour of the mixtures was governed by the effective porosity, which acted as the dominant microstructural parameter. Zeolite was found to cause an increase in the porosity of the final mortars, leading to a reduction in their final strength. In contrast, copper slag was found to reduce the porosity of the mortars. This hypothesis was demonstrated and has enabled clear relationships to be established between composition, porosity, and final strength.

Zeolite, due to its inherent properties, was shown to enhance the w/c ratio, leading to elevated porosities (27–34%), which consequently reduced the mechanical strength at 28 days. While the pozzolanic activity of this zeolite has been demonstrated in other works, it remained undetected in this study due to the exceedingly high w/c ratio. This underscores the necessity for future research to incorporate strategic water management to enhance this reactivity, while concurrently limiting zeolite dosage to a maximum of 25%.

In contrast, the utilisation of copper slag enabled the attainment of lower w/c ratios compared to reference samples (and samples containing zeolite). This is primarily attributable to the vitreous nature of the slag. Beyond reducing porosity, the fine particle size distribution (finer than the fine aggregate) was conducive to achieving a superior mineral structure when amalgamated with natural materials. Consequently, this resulted in enhanced final strengths, optimal for aggregate substitutions of approximately 50%.

The conjunction of both materials is of importance, as it was evidenced that water not absorbed by the slag was instead absorbed by the zeolites. This enabled the materials to complement each other, culminating in the highly promising MZ-8 and MZ-9 mixtures, which exhibit moderate porosity and the highest mechanical strengths (38–39 MPa) among the three groups of mixtures developed. These mixtures maximise the utilisation of recycled aggregate and achieve a substantial reduction in the carbon footprint, thereby aligning with the principles of the circular economy and emissions reduction without compromising mechanical performance.

Finally, this work could be considered to serve as a guide for using highly available materials or industrial by-products, such as zeolites or copper slags, which have stable physical, chemical, and mineralogical characteristics. These materials can become an environmental liability if not properly managed. This promotes a reduction in CO_2_ emissions and the development of the circular economy.

Overall, these findings confirm that the combined use of copper slag and natural zeolite offers a balanced strategy to reduce clinker and natural aggregate consumption while maintaining acceptable mechanical performance. The complementary behaviour observed between both materials provides insight into how water demand and mixture design influence early-age properties, contributing to a more informed use of industrial by-products in mortar production. Future work will extend the evaluation to long-term performance indicators to complete the assessment of the most promising mixtures.

## Figures and Tables

**Table 1:** Mix proportions.

Group	Mixture	Cement		Zeolite		Aggregates		Slags	
		Weight (%)	Mass (g)	Weight (%)	Mass (g)	Weight (%)	Mass (g)	Weight (%)	Mass (g)
REF ^1^	REF	100	450.0	0	0.0	100	1350.0	0	0.0
REF ZEO ^2^	MZ-1	75	337.5	25	112.5	100	1350.0	0	0.0
	MZ-2	60	270.0	40	180	100	1350.0	0	0.0
	MZ-3	45	202.5	55	247.5	100	1350.0	0	0.0
REF Slags	MZ-4	100	450.0	0	0.0	50	675.0	50	675.0
	MZ-5	100	450.0	0	0.0	25	337.5	75	1012.5
	MZ-6	100	450.0	0	0.0	0	0.0	100	1350
ZEO 1	MZ-7	75	337.5	25	112.5	50	675.0	50	675.0
	MZ-8	75	412.5	25	137.5	25	412.5	75	1237.5
	MZ-9	75	412.5	25	137.5	0	0.0	100	1650.0
ZEO 2	MZ-10	60	270.0	40	180.0	50	675.0	50	675.0
	MZ-11	60	330.0	40	220.0	25	412.5	75	1237.5
	MZ-12	60	330.0	40	220.0	0	0.0	100	1650.0
ZEO 3	MZ-13	45	202.5	55	247.5	50	675.0	50	675.0
	MZ-14	45	247.5	55	302.5	25	412.5	75	1237.5
	MZ-15	45	247.5	55	302.5	0	0.0	100	1650.0

^1^ Reference, ^2^ zeolite.

**Table 2:** Composition of materials obtained by X-ray fluorescence.

Compounds in % Weight				
	Cement	Zeolite	Aggregates	Slags
Al_2_O_3_	4.76	9.64	1.55	2.65
CaO	>60	1.20	0.16	1.32
Cr_2_O_3_	0.01	<0.01	<0.01	0.04
Fe_2_O_3_	3.57	1.27	0.61	63.98
K_2_O	1.04	2.14	0.61	0.59
MgO	1.47	1.09	0.09	0.71
MnO	0.05	<0.01	0.01	0.04
Na_2_O	0.30	3.63	0.19	0.26
P_2_O_5_	0.12	<0.01	0.03	0.06
SO_3_	3.24	<0.01	0.02	1.44
SiO_2_	19.86	68.42	95.44	30.32
SrO	0.03	<0.01	<0.01	0.01
TiO_2_	0.25	0.11	0.06	0.20
LOI ^1^	3.77	11.58	0.47	−6.66

^1^ Loss of ignition.

## Data Availability

The original contributions presented in this study are included in the article. Further inquiries can be directed to the corresponding author.
